# FOXA2 in islet biology: Orchestrating pancreatic development and glucose homeostasis

**DOI:** 10.1016/j.gendis.2025.101972

**Published:** 2025-12-09

**Authors:** Ahmed K. Elsayed, Yusra Manzoor, Essam M. Abdelalim

**Affiliations:** a Laboratory of Pluripotent Stem Cell Disease Modeling, Translational Medicine Division, Research Branch, Sidra Medicine, Doha 26999, Qatar; b College of Health and Life Sciences, Hamad Bin Khalifa University (HBKU), Qatar Foundation, Education City, Doha 34110, Qatar

**Keywords:** α-cells, β-cell function, FOXA2, Glucose homeostasis, Pancreatic development

## Abstract

Forkhead Box A2 (FOXA2) is a transcription factor essential for endodermal development and the formation and function of several metabolic organs, including the liver and pancreas. Within the pancreatic lineage, FOXA2 plays a crucial role in orchestrating islet development, maintaining β-cell identity, and regulating genes central to glucose sensing and insulin secretion. This review provides a comprehensive overview of FOXA2's dual role in both developmental and mature stages of pancreatic islets, highlighting its function as a gatekeeper of lineage specification and metabolic homeostasis. We describe FOXA2's dynamic expression patterns during embryogenesis, its regulatory interactions with other key transcription factors, such as PDX1 and NKX6.1, and its influence on chromatin accessibility during islet cell differentiation. Furthermore, we discuss the consequences of FOXA2 dysregulation, including impaired α- and β-cell maturation, loss of functional identity, and contributions to the pathogenesis of diabetes. Insights from mouse models, human stem cell-derived islets, and patient genetics underscore the clinical relevance of FOXA2 in monogenic and complex forms of diabetes. By integrating developmental biology, genomics, and disease modeling approaches, this review highlights FOXA2 as a central regulator connecting pancreatic organogenesis with long-term metabolic control. Understanding FOXA2's regulatory networks may open new avenues for therapeutic strategies aimed at restoring or preserving β-cell function in diabetes.

## Introduction

Diabetes mellitus, a complex metabolic disorder characterized by chronic hyperglycemia, is intricately linked to the development and function of the pancreas, particularly the insulin-producing β-cells.^1^ A sophisticated interplay of signaling pathways, growth factors, and transcription factors (TFs) is pivotal in regulating pancreatic development, orchestrating the differentiation, proliferation, and maturation of both endocrine and exocrine cell populations within the pancreas.^2^ Numerous studies have identified a myriad of TFs that play a divergent role in β-cells development, maturation, and functionality.^3^ Deficiency or sustained loss of these TFs can lead to β-cell defect and the progression of diabetes mellitus.^4^ For example, *Pdx1* deficiency in mice results in hyperglycemia and reduced β-cell proliferation and mass.^5^ Mutations in *PAX4* cause diabetes mellitus, while its overexpression increases β-cell proliferation.^6^ Mutations in *NEUROD1* are linked to maturity-onset diabetes of the young (MODY) and permanent neonatal diabetes.^7^ Understanding the intricate network of conserved TFs and their molecular dynamics is crucial for repairing or replenishing β-cells.

Forkhead box (FOX) proteins constitute a diverse family of TFs that participate in numerous cellular processes, such as development and metabolism. These proteins are essential across different species, affecting cell cycle regulation, epithelial differentiation, placental development, and other vital biological functions. Mutations in *FOX* genes can lead to various human diseases, including cancer, glaucoma, and metabolic disorders.^8^^,^^9^ The *Fox* gene family is an ancient evolutionarily group of homeotic genes named after the fork head (*fkh*) gene in *Drosophila melanogaster*, as its mutation disrupts the head fold involution during embryogenesis, resulting in adult flies with a distinctive spiked head appearance resembling a fork.^10^ Since the discovery of the *fkh* gene in *Drosophila melanogaster*, more than 170 FOX family members have been identified across various species and classified into 19 subfamilies.^11^^,^^12^ The initial discovery phase resulted in a multitude of names assigned by various researchers, leading to significant confusion. To address this and establish a standardized system for studying *Fox* genes, the Fox nomenclature committee proposed a classification system in 2000, based on a phylogenetic analysis of 172 FOX proteins from 14 species.^13^ This system initially divided FOX proteins into 15 classes (FOXA to FOXO) based on similarities in the forkhead domain (FKH domain) and has since been expanded to include four additional classes (FOXP to FOXS), culminating in a total of 19 clades. To further streamline the nomenclature process, a unified designation "FOX" was adopted, followed by a number to identify individual proteins, thereby facilitating clearer communication and more effective research within the scientific community.^14^ The Foxa protein family was initially identified through its DNA binding activity in liver nuclear extracts, which specifically targeted the promoters of the albumin, transthyretin, and α1-antitrypsin genes (*Alb1*, *Ttr*, and Serpina1, respectively).^15^^,^^16^ These FOXA protein members were originally designated as hepatocyte nuclear factor-3 (HNF-3) α, β, and γ. With the introduction of the standardized nomenclature, they were reclassified, and the mammalian FOXA subfamily is now identified as FOXA1 (HNF3α), FOXA2 (HNF3β), and FOXA3 (HNF3γ).^13^ They exhibit a high degree of similarity, with their sequences being 95% identical. While their homology is weaker outside the forkhead box, the N and C termini show the highest conservation. Foxa3 is the least closely related, compared with Foxa1 and Foxa2.^17^ The FOXA subfamily has been extensively studied genetically and biochemically, revealing its crucial roles throughout mammalian life. Its three members are essential from early development through organogenesis to adult metabolism and homeostasis. They are vital for foregut endoderm competence and the development of endoderm-derived organs such as the liver, pancreas, lungs, and prostate.^18^^,^^19^ Postnatally, FOXA proteins regulate glucose and fat metabolism by controlling target genes in the liver, pancreas, and adipose tissue.^17^^,^^20^^,^^21^ Numerous congenital diseases are linked to disruptions in FOXA regulation, with point mutations in these genes contributing to various pathologies. Additionally, the role of FOXA proteins in carcinogenesis has been well-documented.^22^

Foxa2 is a fundamental pioneer TF discovered through a screen of mutations for embryonic lethality.^10^^,^^23^ Initially identified as hepatocellular nuclear factor 3β (HNF3β), Foxa2 is essential for liver development, where it regulates the expression of hepatic genes, maintains bile acid homeostasis, and prevents cholestatic injury by controlling hepatocyte and cholangiocyte growth.^24^^,^^25^ Besides its fundamental role in organogenesis, this review mainly focuses on the role of FOXA2 in pancreatic development and diabetes pathogenesis.

## Structural dynamics, DNA binding, and functional activity of FOXA2 protein

**Figure 1:**
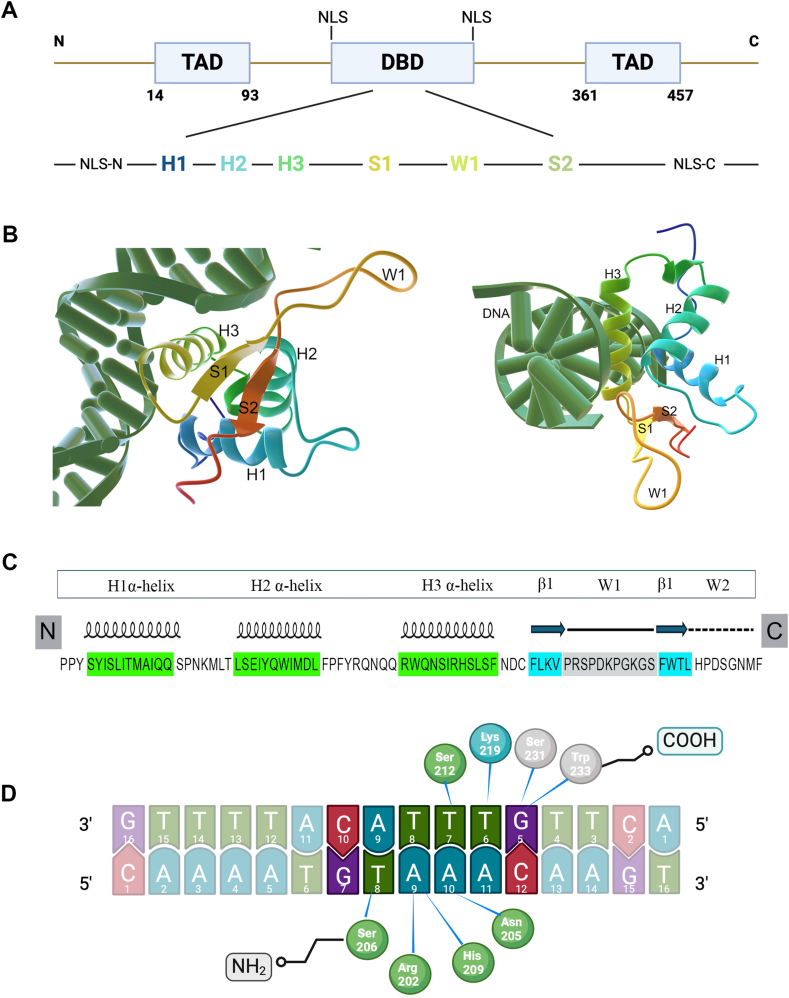
Structural features, DNA interaction, and functional role of FOXA2 protein. **(A)** The FOXA2 protein contains two transcription activation domains (TADs) and a relatively conserved DNA-binding domain (DBD), which comprises a hydrophilic helix core flanked by two wings **(B, C)**. The DBD recognizes its target DNA sequence by inserting the H3 α-helix into the major groove of the DNA backbone, forming several hydrogen bonds with specific amino acids upon binding **(D)**. The crystal structure of FOXA2 is autogenerated by NIH 3D workflows from public source data https://3d.nih.gov/entries/3dpx-017447.

The full-length structure of mammalian FOXA protein consists of two transcription activation domains (TAD) and a relatively conserved 100-residue DNA binding domain (DBD) (between 157 and 257 residues in FOXA2) that is found in all FOX family of proteins and is so known as Forkhead domain (FHD).^26^ FOXA2 transcription activation domain Ⅰ is located at the N-terminal region (14–93 residues) and domain Ⅱ at the C-terminal region (361 and 457 residues) (Fig. 1A). The DNA-bound FKH domain comprises a hydrophilic helix core flanked by two wings, which resemble the butterfly, and so it has also been termed as winged-helix domain. It consists of three α-helices (H1-3) at the N-terminus, three-stranded β-sheets (S1-3), and two wing-like loops (W1 and W2), which are less conserved among FOX proteins.^14^^,^^23^^,^^27^ Structural studies on the FOXA2 DBD have revealed distinct variations that set it apart from other FOX family proteins. While the DBD of FOXA2 retains the typical three α-helices, it uniquely features only two antiparallel β-strands (S1 and S2) and a single winged loop (wing 1) between them (Fig. 1B and C). Moreover, wing 2 is disordered in the crystal structure and not well-defined, but it is believed to be important for optimal DNA binding, as its deletion results in significantly reduced binding affinity.^26^ Another unique characteristic of Foxa2 is the presence of an AKT2/PKB phosphorylation site at the N-terminus of the forkhead domain, a feature not found in other Fox proteins. The functional significance of this phosphorylation site remains controversial and warrants further investigation.^17^ Chromatin immunoprecipitation-sequencing analysis of FOXA2-binding sites across human and mouse adult liver tissues has shown that FOXA2 interacts with a 16-bp double-stranded DNA (5′CAAAATGTAAACAAGA3′) containing a consensus sequence (5′GTAAACA3′) of the FOX family.^26^^,^^28^ Most FOX proteins typically bind to DNA as monomers.^23^^,^^27^ The FOXA2 protein primarily recognizes its DNA sequence through the interaction of its H3 α-helix, which inserts itself into the major groove of the DNA backbone upon binding^29^ (Fig. 1B). This interaction facilitates the formation of multiple hydrogen bonds, with specific amino acids playing key roles. For example, amino acid N205 forms bidentate hydrogen bonds with A10, while H209 interacts with T8’ and A9 through base-specific hydrogen bonds. The phosphate groups of A9, T8, and T7 are also recognized and bound by hydrogen bonds with the amino acids R202, S206, and S212, respectively (Fig. 1D). Alongside these hydrogen bonds, numerous van der Waals contacts also contribute to the stability of the FOXA2-DNA complex.^26^ The wing regions potentially recognize DNA structural features in the areas flanking FOX binding sites.^30^ However, sequence divergence in these regions can lead to differences in DNA binding.^27^^,^^29^^,^^31^^,^^32^ In FOXA2, wing 1 plays a key role in DNA binding, with hydrogen bonds forming between the G5’ phosphate group and S231/W233, and K219 interacting with the T6’ phosphate group.^26^ The N and C-terminal of the FKH winged helix domain contain nuclear localization sequences (NLS). The N-terminal NLS, composed of the amino acid residues KPPYSYISLITMAIQQ, is one of the most conserved regions among FOX proteins.^33^ NLS at the C-terminus comprises LRRQKRFKC basic amino acid residues. The conservation of both N- and C-terminal NLS sequences is essential for specific interactions with proteins involved in the proper translocation of the FOXA2 protein into the nucleus.^33^, ^34^, ^35^

## FOXA2 in embryonic organogenesis: A master regulator

**Figure 2:**
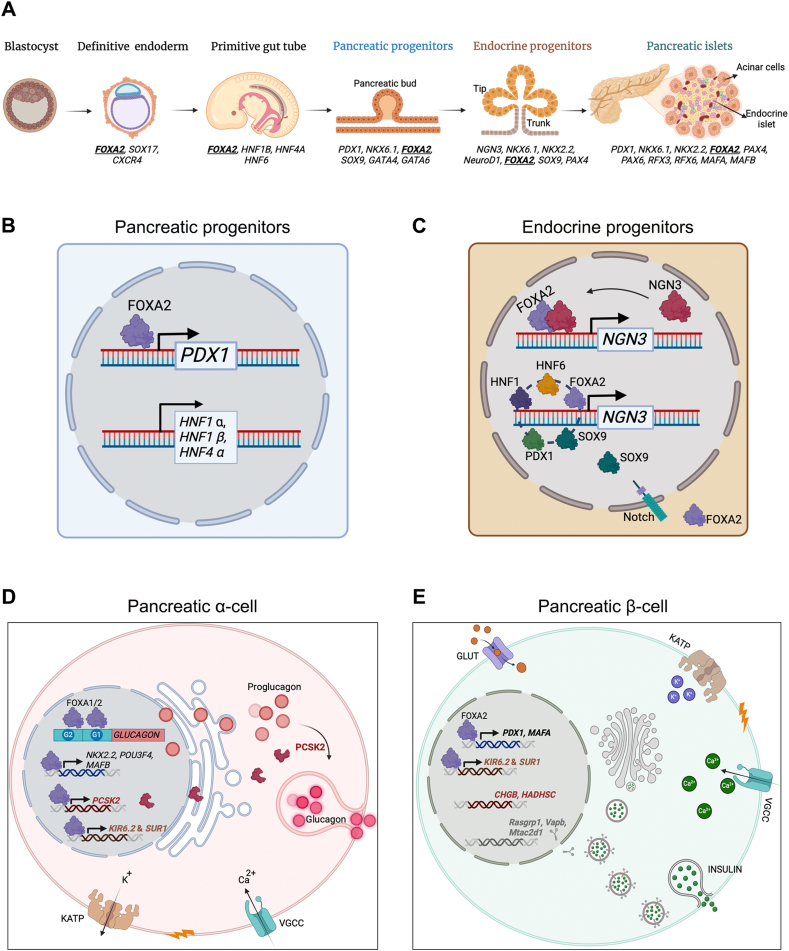
FOXA2 is crucial for pancreatic islet development. **(A)** FOXA2 is expressed during early and late stages of pancreatic development. **(B)** Its expression begins early in the endodermal cells and continues through the various stages of pancreatic development. **(C)** FOXA2 regulates the expression of critical transcription factors required for the development of the endocrine pancreas. FOXA2 is essential for alpha **(D)** and beta **(E)** cell development and function. The figure was created using Biorender (https://biorender.com/).

The expression patterns of FOXA2 indicate that it plays an important role in organ development. Its expression initiates very early in the gastrula stage, specifically in the anterior portion of the primitive node of the embryo. With the expansion of the primitive streak, Foxa2 expression was detected in the anteriorly migrated mesodermal and endodermal cells. In later embryonic stages, its expression becomes restricted to the neural tube, notochord, and the most anterior endodermal foregut, which contributes to the development of crucial organs such as liver, lungs, thyroid, and pancreas.^18^^,^^36^, ^37^, ^38^, ^39^ In humans, FOXA2 expression is initially detected at the fourth week of gestation and persists throughout all stages of pancreatic development (Fig. 2A).^2^^,^^40^ Its early specific expression explains the death of mice lacking *Foxa2* as it demonstrates disability to form the distinct primitive node, subsequently resulting in the loss of the notochord, malformation in the somites and neural tube, causing the death by embryonic day 10 and 11 (E10-11) of the *Foxa2*^*−/−*^ null allelic mouse embryos.^39^ The persistent existence of Foxa2 from the initial embryonic phases to adulthood signifies its fundamental involvement in both the specification and maintenance of tissues derived from the endoderm. Despite the presence of some endodermal cells in the early conditional ablated *Foxa2* mouse model, it fails to form the gut tube and its endodermal derivatives.^41^^,^^42^ Other members of the Foxa family, particularly Foxa1, share similar expression patterns except for the early primitive streak stage. However, Foxa1 expression begins slightly later and is confined to the definitive endoderm and notochord during the headfold stage. It later extends to the entire gut region, including the liver primordium, floorplate of the neural tube, and then persists in adult endoderm-derived organs.^43^^,^^44^ In contrast, Foxa3 is restricted to the hindgut and some skeletal involvement, showing a lesser extent in the embryonic organogenesis, but it is crucial for adult glucose homeostasis.^45^ Studies demonstrate the synergistic roles of FOXA1 and FOXA2 in organ development. Mouse embryos lacking both *Foxa1* and *Foxa2* exhibit a complete deficiency in hepatic specification, with the absence of liver bud formation and expression of hepatic markers.^46^^,^^47^ This synergy contrasts with human development, where *FOXA2* deletion directly impacts hepatocyte and biliary development without compensation from *FOXA1*.^48^ Specific deletion of *Foxa2* in respiratory epithelium resulted in defects in the terminal alveolar differentiation, but the effect is more severe with *Foxa1/2* deletion, leading to immature epithelium and abnormal lung branching.^49^, ^50^, ^51^ This functional redundancy between Foxa1 and Foxa2 is also evident in intestinal cell differentiation, where knockout (KO) studies show disruptions in the enteroendocrine lineages, with a lack of Glp (glucagon-like peptide) expressing cells as well as a severe reduction in the somatostatin and Ppy expressing cells.^52^ In addition to its critical role in endoderm-derived organ development, FOXA2 is essential for certain non-endodermal lineages. Mice with one deleted *Foxa2* allele display Parkinson-like symptoms in 30% of cases, while dual *Foxa1/2* deletion leads to the loss of dopamine transporter (DAT) expression and a reduction in dopaminergic neurons.^53^^,^^54^ During nervous system development, Foxa2 is prominently expressed in the diencephalon and midbrain nuclei, where it plays a pivotal role in the differentiation and maturation of dopaminergic neurons.^17^^,^^55^ FOXA2 also influences motor control and serotonin neuron development via the Sonic Hedgehog (SHH) signaling pathway.^56^^,^^57^ In the prostate, Foxa2 interacts with androgen receptors to regulate prostate-specific gene expression and morphogenesis.^58^ Our review primarily focuses on the role of the *FOXA2* gene in pancreatic development and its association with diabetes mellitus. These aspects will be highlighted and elaborated upon in the subsequent sections.

## FOXA2: A key regulator of pancreatic development and cell fate specification

FOXA2 plays a pivotal role in pancreatic development, functioning as a key TF that orchestrates gene expression during critical stages of pancreatic cells' embryogenesis. Its expression is initiated early in the bud outgrowth phase, where it is localized in pancreatic endodermal cells.^17^^,^^59^ FOXA2 is indispensable for the expansion of the pancreatic progenitor pool at the onset of cellular differentiation and is essential for ensuring the proper development of endocrine pancreas precursors^60^ (Fig. 2A). Due to the early embryonic lethality associated with homozygous *Foxa2* deletion, conditional KO models have been employed to elucidate its function in endoderm-derived organs. Transgenic mice harboring a floxed *Foxa2* allele (*Foxa2*^*loxP/loxP*^) crossed with strains expressing Cre-recombinase under the control of Foxa3, Pdx1, or Insulin promoters (*Foxa2*^*loxp/loxp*^;Foxa3Cre, and *Foxa2*^*loxp/loxp*^;Pdx1Cre, and *Foxa2*^*loxp/loxp*^;InsCre) have provided crucial insights into the role of FOXA2 in pancreatic development^46^^,^^61^ (Table 1). While these mouse models have significantly advanced our understanding, they fall short of fully recapitulating human development. To bridge this gap, human pluripotent stem cell (hPSC) models deficient in *FOXA2* have been utilized, offering deeper insights into its pivotal role in human pancreatic development.^18^^,^^62^^,^^63^ These models emphasize the significance of FOXA2 in the intricate regulatory networks that govern pancreatic organogenesis and highlight its potential implications for understanding developmental disorders and diseases such as diabetes (Table 1).

**Table 1:** Targeted deletions, conditional null alleles, and compound mutation of *Foxa2* in animal and human models for pancreas development.

Genotype	Type	Phenotype	Reference
*Foxa2*^*−/−*^	Null alleles (embryonic)	Significant abnormalities observed in the node, notochord, neural tube, and gut tube, leading to mortality by embryonic day 10–11	39
*Foxa2*^*+/−*^	Null alleles (embryonic)	Reduced glucose uptake and glycolysis, along with increased adiposity, when on a high-fat diet	147
*Foxa2*^*LoxP/LoxP*^;Foxa3*.Cre*	Conditional null mutation (endoderm)	Mortality observed between postnatal days 0 and 5, accompanied by hypoglycemia and hypo-glucagonemia	64
*Foxa1*^*−/−*^;*Foxa2*^*LoxP/LoxP*^;Foxa3*.Cre*	Compound and conditional null mutation (endoderm)	Mortality occurs between embryonic days 9.5 and 10.5, with a loss of liver specification.	46
*Foxa2*^*+/LoxP*^;pdx1*.Cre*	Conditional (pancreatic progenitors)	A single allele of FOXA2 is adequate for pancreatic induction and the maintenance of normal metabolic functions	59
*Foxa2*^*LoxP/LoxP*^;pdx1*.Cre*	Conditional (pancreatic progenitors)	Significant decrease in pancreatic mass	59
*Foxa1*^*LoxP/–*^;*Foxa2*^*LoxP/LoxP*^;pdx1*.Cre*	Conditional and compound mutation (pancreatic progenitors)	Severe pancreatic hypoplasia accompanied by pronounced hyperglycemia.	59
*Foxa2*^*LoxP/LoxP*^;Ins*.Cre*	Conditional null mutation (β-cells)	Mortality observed between postnatal days 9 and 12, accompanied by hypoglycemia and hyperinsulinism	61,84
*Foxa2*^*loxP/loxP*^;Pdx1*-CreERT2*	Conditional null mutation (β-cells)	Increased docking of insulin granules, along with elevated levels of cyclic AMP (cAMP) and calcium (Ca^2+^) oscillations, resulting in enhanced exocytosis and hyperinsulinism	32
*Foxa2*^*−/−*^	Knockdown (mature pancreatic α-cells)	Impaired α cell specification, leading to hypo-glucagonemia and hypoglycemia	64,82
*Foxa2*^*LoxP/LoxP*^;Alb*.Cre*	Conditional null mutation (hepatocytes)	Normal	46
*Foxa2*^*LoxP/LoxP*^;Alfp*.Cre*	Conditional null mutation (hepatocytes)	Reduced the induction of gluconeogenic enzymes	142
*Foxa1*^*−/−*^;*Foxa2*^*LoxP/LoxP*^	Conditional and compound mutation (hepatocyte)	Mortality occurs between embryonic days 9.5 and 10.5, accompanied by the loss of liver specification	46
*Foxa2*^*VenusKO*^	Knockout mutation (mouse ESCs)	Significant defect in the differentiation of definitive endoderm	65
*FOXA2*^*+/−*^	Haploinsufficiency (hiPSCs)	Impairment in endodermal lineage formation, characterized by down-regulation of genes involved in pancreatic development	62
*FOXA2*^*−/−*^	Knockout (hiPSCs)	Impairment in endodermal lineage formation, associated with the down-regulation of pancreatic development genes	62
*FOXA2*^*−/−*^	Knockout mutation (hESCs)	Moderate impairment in the differentiation of definitive endoderm	63
*FOXA1/2*^*−/−*^	Knockout mutation (hESCs)	Slight defect in the differentiation of definitive endoderm	18

### Role of FOXA2 in the development of definitive endoderm

FOXA2 is essential for proper endoderm and foregut development, as its absence leads to significant developmental defects and malformations with serious consequences and malfunctions. In a study using *Foxa2*^*loxp/loxp*^;Foxa3Cre mice, specific deletion of *Foxa2* in the endoderm revealed normal Foxa2 expression in the floor plate and notochord, but a complete absence in the midgut. Meanwhile, its expression is significantly reduced in the foregut endoderm during early development and entirely absent during later stages of pancreatic specification. This lack of Foxa2 expression was confirmed in other endoderm-derived organs, including the liver, stomach, and intestine.^64^ The Cre recombinase mouse model system in this model is driven by the *Foxa3* promoter, which is expressed slightly later than Foxa2. This temporal difference allows Foxa2 to contribute to anterior endodermal processes like gut invagination and formation, making this model less suited for studying FOXA2's role in early endoderm specification. Knocking out *Foxa2* in mouse ESCs (mESCs) significantly impairs the *in vitro* definitive endoderm differentiation, as evidenced by the marked reduction in key endodermal markers such as Sox17, retention of pluripotency genes, and improper activation of endodermal signaling pathways.^65^ Similarly, haploinsufficiency and complete deletion of the *FOXA2* gene in hiPSCs (*FOXA2*^*+/−*^ hiPSCs and *FOXA2*^*−/−*^ hiPSCs) disrupts endodermal lineage formation, while retaining pluripotency markers after differentiation.^62^ The early lethality of the non-conditional *Foxa2* KO mouse model and the failure of endodermal differentiation of *FOXA2*^*−/−*^ PSCs underscore FOXA2's master regulatory role in endodermal germ layer specification. However, other hESC models revealed that *FOXA2*^*−/−*^ and *FOXA1/2*^*−/−*^ have a lesser impact on the definitive endoderm differentiation, with SOX17 expression comparable to control cell lines.^18^^,^^63^ Importantly, Foxa2 can directly regulate the transcription of Gata4 by binding to its enhancer.^66^ Gata4 is a key TF that controls both early endoderm development and later stages of pancreatic differentiation.^67^

### FOXA2 drives the pancreatic progenitor specification and development

FOXA2 is crucial for the early specification of pancreatic primordia, which is implicated in pancreatic bud initiation, expansion, and the subsequent maintenance and regulation of mature, differentiated pancreatic islet cell types^17^^,^^59^^,^^68^^,^^69^ (Fig. 2B). It has been demonstrated that Foxa2 binds the regulatory regions of *Pdx1*, a key gene essential for pancreas formation. Chromatin immunoprecipitation-sequencing studies revealed the binding of Foxa2 to four areas in the *Pdx1* enhancer (I-IV) in the fetal pancreas, with the strongest affinity for region IV, located 6.4 Kb upstream of the *Pdx1*coding region.^59^ Despite this regulatory evidence of Foxa2 to *Pdx1* at the chromatin level, conditional deletion of *Foxa2* in the endoderm (*Foxa*^*loxp/loxp*^;Foxa3*Cre* mice) still allows the proper pancreatic bud formation with normal expression of Pdx1^64^ suggesting that Foxa2 may not be essential for the onset of pancreatic development in mice and may be compensated by other factors. Interestingly, when *Foxa2* is deleted specifically in the pancreatic primordia using Pdx1Cre transgenic mice, a single allele of *Foxa2* (*Foxa*^*loxp/+*^;Pdx1Cre) is sufficient for the induction of pancreas development with normal metabolic activities. Meanwhile, the homozygous deletion *Foxa2*^*loxp/loxp*^;Pdx1Cre significantly reduces pancreatic mass, with the presence of remaining pancreatic cell types, which is explained to be under the compensatory effect of Foxa1. The compound deletion of *Foxa2* and *Foxa1* (*Foxa1*^*loxp/+*^;*Foxa2*^*loxp/loxp*^;Pdx1Cre) led to severe pancreatic hypoplasia, complete loss of Pdx1-expressing cells, pronounced severe postnatal hyperglycemia, and early death.^59^ This highlights the compensatory relationship between Foxa2 and Foxa1 in mouse pancreatic development. While mouse models indicate some compensation by Foxa1, hPSC models reveal a more critical requirement for FOXA2, which is indispensable for proper pancreatic progenitor differentiation, with gene dosage playing a significant role. The complete absence of *FOXA2* in hPSCs severely impairs the formation of pancreatic progenitors with extensive loss of PDX1 and NKX6.1 expression, the key regulators of pancreatic primordia.^18^^,^^62^^,^^63^ The pancreatic progenitors derived from *FOXA2*^*−/−*^ hiPSCs exhibit down-regulation of genes involved in pancreas development and diabetes while up-regulating genes associated with lipid metabolism and nervous system development.^62^^,^^70^ Heterozygous deletion of *FOXA2* moderately affects the pancreatic progenitor's differentiation, but to a lesser extent than complete KO models.^62^ Interestingly, in human models, *FOXA1* compensation is absent, as it is markedly reduced in FOXA2-deficient pancreatic progenitors.^62^ FOXA2 also positively regulates other hepatocyte nuclear factors (HNFs), including HNF-4α, HNF-1α, and HNF-1β, along with their downstream targets.^71^^,^^72^ In *Foxa2*-null embryoid bodies, the expression of *Hnf-4α* and its downstream targets, such as apolipoproteins AI, AII, AIV, B, CII, and CIII; aldolase B; l-pyruvate kinase; and transthyretin, is significantly reduced, alongside a decrease in *Hnf-1α* expression.^71^ In mouse studies, HNF1β is shown to be essential for the specification, growth, and differentiation of the embryonic pancreas, and mutations in HNF1β often lead to pancreas hypoplasia in humans.^73^ HNF1α and HNF4α are vital for β-cells function, with HNF1α regulating both β-cell function and growth by controlling target genes such as *Glut2*, *Hnf4α*, pyruvate kinase (*Pklr*), collectrin, and hepatocyte growth factor activator.^72^^,^^74^

It has been reported that the intensity of Foxa2 expression in the pancreatic bud epithelium (Pdx1+) initiates the segregation of the multipotent progenitors into Pdx1+/Foxa2+ ^high^ population, which will be committed to endocrine progenitors; meanwhile, the other population, Pdx1+/Foxa2+ ^low^, will be the precursors of the acinar and ductal cells. This indicates that varying levels of FOXA2 expression, as a result of its transcriptional activities, define different pathways of cellular commitment and specification^60^ and this differential expression plays a pivotal role in guiding the commitment of the pancreatic progenitor cells to differentiate into an endocrine cell (Fig. 2C). Interestingly, Gao and colleagues demonstrated that a single allele of Foxa2 is sufficient to induce Pdx1 expression and sustain normal pancreatic function and islet development.^59^

### Role of FOXA2 in shaping the pancreatic endocrine lineage

During the initial stages of pancreatic development, FOXA2 is expressed in the majority of pancreatic progenitor cells and later becomes restricted to the endocrine progenitors, with reduced expression levels in the exocrine and ductal lineages.^75^ In animal models, early endodermal deletion of *Foxa2* specifically impacts α-cells, glucagon (Gcg)-producing cells, leading to a marked decrease of glucagon expression, while other endocrine and acinar cells remain unaffected. Subsequently, the offspring from this model exhibit severe hypoglycemia, disorganized islets, and die shortly after birth due to metabolic instability.^64^ On the other hand, the later deletion of *Foxa2* at the pancreatic specification stage using Pdx1.Cre transgenic mice impair the development of all the endocrine and exocrine cells, resulting in severely hypoplastic islets, and the mutant mice died due to severe hyperglycemia. These mutant mice exhibit near-complete loss of critical TFs such as Pdx1, Nkx6.1, Nkx2.2, Pax6, and Isl1. At the same time, Gao and colleagues exposed that a single allele of *Foxa2* is sufficient to induce Pdx1 expression and sustain normal pancreatic function and islet development.^59^ In hiPSC models, haploinsufficiency and KO of *FOXA2* is associated with significant alterations and reduction of most of the key regulators of endocrine specification, including *NKX6.1*, *NOTCH*, *NGN3*, *NEUOROD1*, *NKX2.2*, and *PAX4*.^62^ Foxa2 has been shown to regulate the endocrine lineage through its direct cooperation with Neurog3, as Foxa2 synergizes with Neurog3 to activate its own promoter.^76^ Neurogenin3 (Neurog3) is a master regulator for pancreatic endocrine commitment.^77^ Foxa2 can control Neurog3 through its upstream effect on Pdx1, which is a part of the cross-regulatory network up-regulating Neurog3 (Pdx1, Hnf1b, Hnf6, and Foxa2).^78^ Pdx1 participates directly in the activation of Neurog3, leading to up-regulation of Neurod1 that enhances the endocrine specification.^79^ In the absence of FOXA2, NOTCH signaling is reduced,^62^ which is required with an intermediate expression level to activate Sox9 and subsequent up-regulation of Neurog3.^80^ Consistent with findings from the *Foxa2*^*−/−*^ *Pdx1*Cre model, the human FOXA2 KO hiPSCs model failed to generate the main endocrine cells of the pancreatic islet stage of differentiation, with a near complete loss of INS and GCG expression.^62^

### FOXA2 role in the specification and functionality of mature glucagon-producing α-cells

In the developing pancreas, pancreatic precursor cells are first specified to become endocrine progenitor cells and then are regulated by the combinatorial effects of TFs to differentiate into α-, β-, δ-, pancreatic polypeptide, or ε-cells. These cells are deemed "mature" once they begin secreting their respective hormones.^81^ Mouse model demonstrated that Foxa2 is not strictly required for the emergence of the first wave of glucagon-positive cells.^64^ However, the terminal differentiation and maturation of glucagon-producing α-cells are dependent on Foxa2, as its absence is accompanied by a reduction of mature α cells, with severe hypoglucagonemia and hypoglycemia.^64^ Specific targeting of the mature α-cells through siRNA knockdown of *Foxa2* in primary pancreatic α-cell lines of rodents revealed that it affects the *Gcg* expression, as well as the genes involved in α-cell differentiation and function.^82^ Foxa2 silencing leads to reduced expression of *Gcg*, *MafB*, *Pou3f4*, and *Nkx2.2*, which are responsible for α-cells specification, as well as *Pcsk2* (prohormone convertase), *Kir6.2*, and *Sur1* (subunits of the K^+^-ATP channel), which are involved in the enzymatic activity of Gcg processing and secretory machinery and the functionality of α-cells. Foxa2 has a direct binding to the promoters of *Nkx2.2*, *Kir6.2*, and *Sur1*^82^, ^83^, ^84^ as well as *Pou3f4*, *Gipr*, and *Isl1*.^82^ Foxa2 is crucial for activation of the *Gcg* gene through its interaction with the G1 and G2 elements of its promoter, and *Foxa2* deficiency is accompanied by a decrease in *Gcg* secretion^82^^,^^85^ (Fig. 2D).

### FOXA2 in insulin-producing β-cells

**Figure 3:**
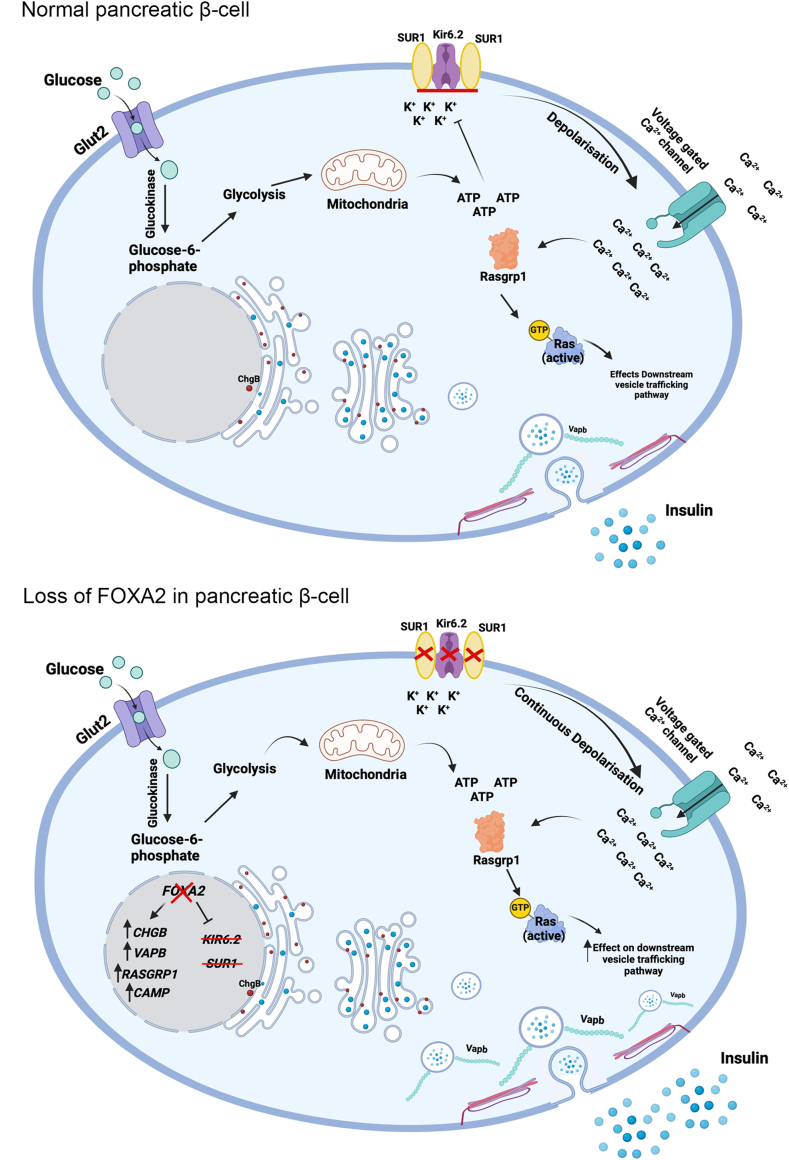
FOXA2's role in β-cell function. FOXA2 plays a critical role in controlling insulin secretion by directly regulating the ATP-dependent K^+^ channel subunits Kir6.2 and Sur1. It also influences several genes involved in secretory granule biogenesis, vesicle trafficking, and exocytosis, such as chromogranin B (ChgB), membrane targeting [tandem] C2 domain containing 1 (Mtac2d1), vesicle-associated membrane protein-associated protein B (Vapb), and RAS guanyl releasing protein 1 (Rasgrp1). In the absence of FOXA2, these targets are up-regulated, leading to an increase in docked insulin granules, peripheral accumulation, and heightened exocytosis, which may contribute to the hyperinsulinism phenotype seen in FOXA2 deficiency. The figure was created using Biorender (https://biorender.com/).

FOXA2 plays a critical role not only in pancreatic development and endocrine lineage specification but also in the functional maturation and maintenance of insulin-producing β-cells (Fig. 2E). Animal and human studies have shown that pancreatic islet β-cells are severely affected and reduced in the absence of FOXA2.^59^^,^^62^ Conditional ablation of Foxa2 in adult β-cells of transgenic mice, using either Insulin- or Pdx1-driven Cre recombinase, has revealed key insights into its role in mature islets.^32^^,^^61^^,^^86^ Although islet architecture appears grossly normal in these models, FOXA2 deficiency leads to abnormal endocrine cell organization and a mild reduction in β-cell mass.^61^ Despite no significant changes in insulin or glucagon content, or in β-cell proliferation and apoptosis, Foxa2-deficient islets exhibit a marked functional phenotype. They show increased insulin secretion at low glucose levels and impaired suppression of insulin release when glucose levels fall, leading to dysregulated glucose-stimulated insulin secretion and subsequent hyperinsulinemia and hypoglycemia. This suggests that FOXA2 is dispensable for insulin biosynthesis but essential for proper stimulus-secretion coupling.^61^ Mechanistically, FOXA2 directly regulates critical components of the insulin secretory pathway. It modulates the expression of the ATP-sensitive K^+^ channel subunits Kir6.2 and Sur1, which are vital for glucose-sensing and insulin release, while having minimal impact on Glut2, Gck, or Gdh.^61^ Furthermore, FOXA2 influences the expression of genes involved in vesicle trafficking and exocytosis, such as *Chgb*, *Mtac2d1*, *Vapb*, and *Rasgrp1*,^32^ leading to excessive accumulation of docked insulin granules in FOXA2-deficient β-cells. Altered intracellular messengers, including elevated cyclic AMP (cAMP) levels and dysregulated calcium oscillations, further exacerbate the secretory phenotype^32^ (Fig. 3). Transcriptomic analyses have also identified FOXA2 as a regulator of metabolic genes. Notably, *Hadhsc*, which encodes the enzyme SCHAD involved in fatty acid oxidation, is down-regulated in Foxa2-deficient mouse islets.^84^ Its reduced expression may contribute to lipid accumulation and enhanced insulin secretion independent of glucose, a feature linked to hyperinsulinism.^87^, ^88^, ^89^ Moreover, one of the most important regulators of carbohydrate metabolism and glucose-responsive genes, *Chrebp* or Mlxipl (carbohydrate response element-binding protein), is directly regulated by Foxa2 through binding its cis-regulatory regions.^86^ In addition to its regulatory role of PDX1 during pancreatic development, FOXA2 also specifically activates PDX1 in β-cells by binding to its promoter region. This activation is crucial for maintaining β-cell identity and function, as PDX1 is a key TF involved in insulin gene expression and β-cell maturation. The direct interaction between Foxa2 and the Pdx1 promoter underscores the importance of FOXA2 in the precise regulation of genes essential for β-cell functionality and insulin secretion.^90^, ^91^, ^92^ The MafA is essential for islet β-cell function and exhibits a unique expression pattern specific to pancreatic cell types.^93^ Foxa2 regulates the islet β-cell-specific MafA expression through binding to the region 3 (R3) of its cis-regulatory region with Pdx1 and Nkx2.2.^94^ *FOXA2*^*−/−*^ hiPSC model demonstrated absence of insulin content in the derived islet stage.^62^

While FOXA2 is well-known for its role in pancreatic β- and α-cell differentiation, its involvement in δ-cell (somatostatin-producing cell) development remains less well characterized. Emerging evidence suggests a potential regulatory role. In the gastrointestinal tract, dual deletion of *Foxa1* and *Foxa2* significantly reduces somatostatin-expressing D-cells and lowers *Sst* transcript levels, indicating that Foxa TFs cooperatively regulate somatostatin-producing lineages.^52^ Although direct studies in pancreatic δ-cells are limited, FOXA2 may influence δ-cell specification indirectly. Further investigation using δ-cell–specific FOXA2 KO models is needed to clarify its functional significance in this lineage.

### Role of other FOX family proteins in pancreatic development and potential interplay with FOXA2

Several FOX TFs, including members of the FOXO, FOXM, and FOXP subfamilies, play key roles in pancreatic development and islet cell function. These factors regulate endocrine differentiation, cell cycle progression, and stress responses, often intersecting with FOXA2 at critical transcriptional nodes. The FOXO family of transcription factors regulates key cellular processes, including cell cycle progression, metabolism, and longevity. In mammals, four FOXO isoforms are expressed: FOXO1, FOXO3a, FOXO4, and FOXO6.^95^ FoxO1, the earliest identified and most studied member of the FoxO family, is a key TF expressed in insulin-target tissues and pancreatic β-cells.^96^ During embryonic development, it is broadly expressed in the pancreas, but becomes restricted to β-cells in adulthood, where it remains inactive under normal physiological conditions and is activated by stressors such as oxidative stress and hyperglycemia.^96^^,^^97^ FoxO1 regulates glucose and lipid metabolism, insulin resistance, and β-cell proliferation, differentiation, and apoptosis.^98^ Under oxidative stress, FoxO1 enhances the expression of antioxidant enzymes like SOD and CAT, promoting β-cell survival. However, under severe stress, it induces pro-apoptotic genes, contributing to β-cell dysfunction.^99^, ^100^, ^101^ It exerts a multifaceted regulatory influence on both the differentiation and dedifferentiation processes of pancreatic β-cells.^96^ Under conditions of stress or insulin deficiency, nuclear FoxO1 suppresses the expression of progenitor markers such as Neurog3, while enhancing β-cell maturity genes including *MafA* and *NeuroD1*, thereby preserving the differentiated state of β-cells.^102^, ^103^, ^104^ A key mechanistic link between Foxa2 and FoxO1 is their competitive interaction at the Pdx1 promoter. Both TFs bind overlapping cis-regulatory elements but with opposing effects. Foxa2 activates *Pdx1* to support β-cell function, whereas FoxO1 represses its expression.^105^^,^^106^ Nuclear localization of FoxO1 displaces Foxa2 from the promoter, leading to repression of Pdx1 transcription, inhibition of β-cell proliferation, and potential dedifferentiation. At the same time, FoxO1 promotes the expression of MafA and NeuroD1, serving a protective role against β-cell failure.^103^^,^^106^ While some studies have shown that overexpression of FoxO1 in β-cells reduces Pdx1 transcription and impairs insulin secretion,^107^ other findings suggest their expression is not strictly antagonistic. For example, Habibe et al reported that silencing of FHL2 in MIN6 cells led to elevated Pdx1 expression, even in the presence of nuclear-localized FoxO1.^108^

Beyond the antagonistic interplay between FOXA2 and FOXO1, additional members of the FOX TF family, including FOXM1 and the FOXP subfamily, have emerged as critical regulators of pancreatic development and islet cell function.^109^ FoxM1, a key regulator of cell cycle progression, plays a pivotal role during pancreatic organogenesis by driving the proliferation of both endocrine and exocrine progenitor cells.^109^^,^^110^ FoxM1 directly activates the transcription of genes essential for G1/S and G2/M phase transitions, as well as mitotic progression and cytokinesis, including *Cdc2*5 A/B/C, *CyclinA/D2*, *Cdk1/2*, *AuroraB*, *Plk1*, and *Survivin*.^109^^,^^110^ Conditional ablation of *Foxm1* in the pancreas resulted in profound defects in tissue expansion, affecting both the endocrine and exocrine compartments, decreased insulin content and β-cell mass, and impaired glucose intolerance.^111^ In β-cells, FoxM1 expression also declines with aging, correlating with reduced regenerative potential. Remarkably, reactivation of FoxM1 in aged β-cells restores their proliferative capacity to levels comparable to young β-cells, indicating its therapeutic promise for β-cell regeneration in diabetes.^112^ Similarly, members of the FoxP subfamily, FoxP1, FoxP2, and FoxP4, exhibit redundant but critical functions in islet development. These TFs are broadly expressed in all major islet cell types, including α-, β-, and δ-cells, throughout pancreatic development and into maturity.^113^ Genetic ablation studies using a triple conditional KO model (Foxp1/2/4 cKO) in endocrine progenitors revealed severe postnatal hypoglycemia and hypoglucagonemia due to an approximately 85 % reduction in α-cell mass, while β-cell mass remains largely unaffected.^113^ The α-cell loss was attributed to impaired postnatal proliferation, characterized by decreased Ki67 expression, down-regulation of cell cycle activators (*Ccna2*, *Ccnb1*, *Ccnd2*), and increased expression of the cell cycle inhibitor *Cdkn1a*. Moreover, the remaining α-cells exhibited dysfunctional glucagon secretion, further exacerbating hypoglycemia. These defects persisted into adulthood, indicating a sustained requirement for FOXP1/2/4 in α-cell maintenance and functional integrity.^113^

## Epigenetic regulation of pancreatic specification mediated by FOXA2

**Figure 4:**
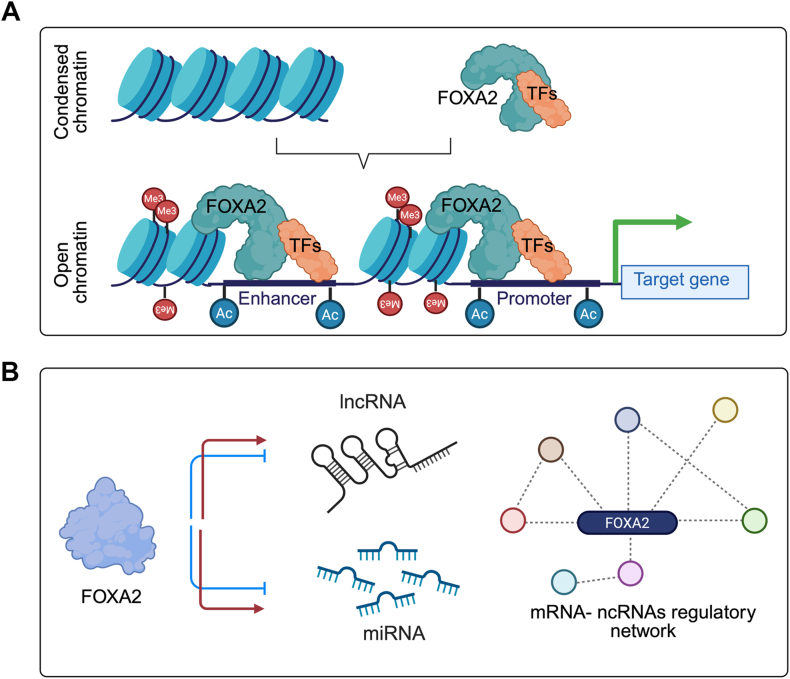
Epigenetic role of FOXA2 in pancreatic specification. FOXA2 acts as a pioneer transcription factor, similar to histone linker proteins, helping to displace linker histones from condensed chromatin. This process enables the binding of other transcription factors to promoter and enhancer regions of target genes and recruits methylases, demethylases, and additional factors essential for gene expression **(A)**. FOXA2 significantly influences non-coding RNA (ncRNA) profiles, particularly the regulatory pathways critical for pancreatic differentiation and β-cell function **(B)**. The figure was created using Biorender (https://biorender.com/).

TFs drive lineage-specific transcription by binding to gene regulatory elements on the nucleosome-free DNA. Unlike most TFs, pioneer TFs can bind to their target sequences on nucleosomal DNA. Once bound, it opens the chromatin, enabling the non-pioneer TFs to activate gene regulatory elements.^114^^,^^115^ FOXA2's pioneering function is largely due to the structure of its forkhead domain, which resembles histone linker proteins H1 and H5. This structural similarity enables FOXA2 to displace histones H3 and H4 in the compacted chromatin and facilitate binding of other TFs to promoters/enhancer regions of target genes and further recruits methylases, demethylases, and other factors needed for gene expression^71^^,^^116^^,^^117^ (Fig. 4A). The unique chromatin-opening ability of FOXA2 has been demonstrated in several studies. For instance, Foxa2 has been shown to bind to the enhancer region of the rat albumin (*Alb*) gene, even when it is compacted into nucleosomes.^28^ Furthermore, FOXA2, along with FOXA1, is essential for chromatin remodeling during pancreatic fate specification.^118^^,^^119^ This capacity to destabilize nucleosomes may be attributed to FOXA2's strong DNA-bending ability.^29^ In addition to activating gene expression, FOXA2 can also repress genes by interacting with regulatory proteins, blocking their transcription by inhibiting their ability to bind DNA.^120^ This dual role in both activation and repression highlights FOXA2's versatility in regulating key developmental pathways across various tissues and organ systems. The mechanisms by which pioneer TFs initiate the opening of surrounding chromatin remain poorly understood. The Zaret group demonstrated that Foxa proteins can open highly compacted chromatin *in vitro* independently of the SWI/SNF chromatin remodeling complex.^121^ This ability is attributed to the c-terminal domain of Foxa, which interacts with core histones H3 and H4^115^^,^^122^. Foxa proteins have been shown to alter chromatin structure in an ATP-independent manner *in vitro* and displace linker histone H1 *in vivo*. Additionally, Foxa1/2 can recruit nucleosome remodeling complexes (Nap1l1/SWI/SNF/INO80), facilitating nucleosome eviction at their binding sites.^121^^,^^123^ A recent study demonstrated that ten-eleven-translocation methylcytosine dioxygenases 1 (TET-1), an enzyme that catalyzes DNA demethylation, is required for FOXA2 recruitment to promoters/enhancers for chromatin remodeling and activity.^119^ During endoderm differentiation, Foxa2 is essential for chromatin opening and the recruitment of active histone modifications. Increased chromatin accessibility is observed at binding sites where Foxa2 collaborates with other TFs, such as Gata4. In ESC-derived endoderm, the co-expression of Foxa2 and Gata4 leads to enhanced chromatin accessibility at their shared binding sites.^65^ Previous research has demonstrated that FOXA2 is recruited to primed enhancers marked with H3K4me1 in endodermal lineages with enrichment of FOXA binding sites in open chromatin regions linked to the initiation of pancreatic differentiation.^124^ An increasing level of H3K4me1 has been reported in human fibroblasts after FOXA2 overexpression.^125^ ATAC-seq (assay for transposase-accessible chromatin with sequencing) analysis of various stages of *in vitro* pancreatic differentiation of hESCs, including definitive endoderm, posterior foregut, and pancreatic progenitors, revealed dynamic chromatin landscape changes. FOXA2 motifs are associated with open chromatin sites throughout all transitions, with the most significant enrichment observed during the posterior foregut-to-pancreatic progenitor transition.^63^ These findings are corroborated by motif enrichment analysis using the Hypergeometric Optimization of Motif Enrichment (HOMER) algorithm.^126^ The deletion of *FOXA2* leads to a reduction in open chromatin accessibility, as well as diminished H3K4me1 and H3K27ac signals. This also impairs the recruitment of GATA6 to pancreatic enhancers.^63^ These findings indicate that FOXA2 is necessary for establishing the primed enhancer state at various stages of pancreatic differentiation and development and is crucial for the proper recruitment of GATA6 to pancreatic enhancers.^63^ miRNA epigenetic modifiers are known to suppress their target mRNAs, hence down-regulating the respective gene. Studies have been carried out where the roles of different miRNAs have been linked to regulate essential genes required for pancreatic differentiation.^127^, ^128^, ^129^ The mode of inhibition of the miRNA on their target genes is mostly by either mRNA de-adenylation activation, which leads to mRNA degradation, or translational repression.^130^^,^^131^ Long non-coding RNAs (lncRNAs) as epigenetic modifiers are essential for controlling cell specification and development.^132^ Several specific lncRNAs in pancreatic islets have been mapped close to TFs that regulate pancreas embryogenesis and β-cell development.^133^ In our recent studies, we showed that deletion of *FOXA2* leads to a significant dysregulation in the map of ncRNAs including lncRNAs and miRNAs (Fig. 4B). Prediction analysis detected several miRNAs to target the significantly down-regulated genes at the pancreatic progenitor and the pancreatic islet stages, which affecting the differentiation efficiency and pancreatic development^70^ such as *hsa-miR-184*, *has-miR-9-5p*, *hsa-miR-199a-3p*, *hsa-miR-767-5p*, *hsa-miR-367-3p*, *hsa-miR-625-5p*, *hsa-miR-194-5p*, and *hsa-miR-92a-2-5p*. Moreover, our miRNA-seq data identified several differentially expressed miRNAs (DEmiRs) previously linked to epigenetic modifications in various tissues. The absence of FOXA2 disrupts not only miRNAs that regulate genes involved in pancreatic development and function, but also miRNAs associated with epigenetic modifications, potentially altering histone marks and DNA accessibility for transcription.^70^ We observed that FOXA2 dysregulation disrupts lncRNA profiles during the pancreatic progenitor and β-cell stages. These lncRNAs are strongly associated with critical pancreatic genes and TFs, including *MEG3*, *H19*, *ZNF667-AS1*, *LINC00543*, *LINC00261*, *AC097639.1*, *AL035661.1*, *SLC25A25-AS1*, *U73166.1*, *ZNF790-AS1*, *MNX1-AS2*, and *AC091563.1*. This suggests that the impairment in pancreatic development seen without FOXA2 is linked to significant changes in lncRNA expression.^134^ These findings, together with prior research, suggest that FOXA2 dysregulation may critically impact the development and function of pancreatic islet cells through the alteration of ncRNA profiles. This alteration in ncRNA expression patterns may lead to islet cell malfunction, resulting in impaired insulin production and secretion.^135^ Consequently, these molecular changes could increase the susceptibility to diabetes. Understanding the role of FOXA2 in ncRNA regulation within pancreatic biology may reveal new therapeutic targets for diabetes management and prevention.

## The role of FOXA2 in metabolism and glucose homeostasis regulation

**Figure 5:**
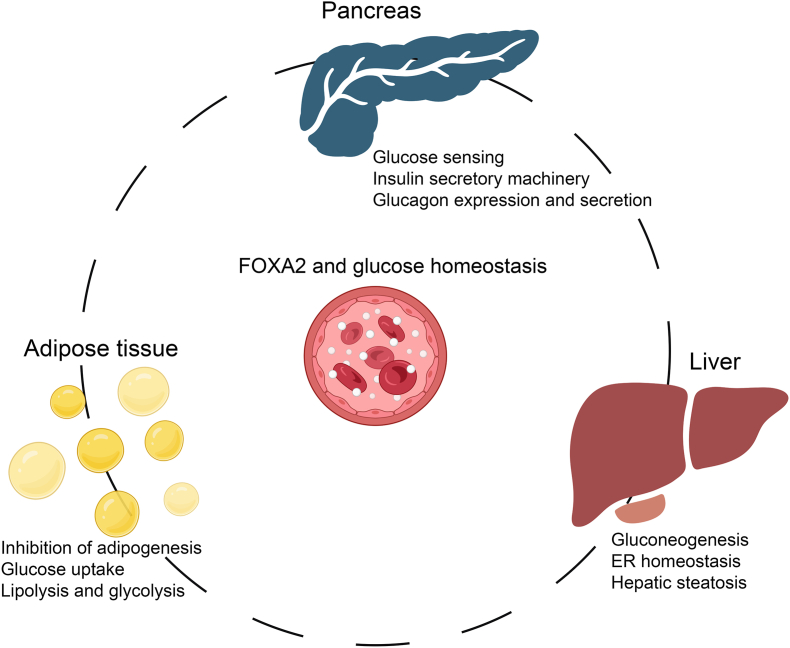
FOXA2 is a key regulator of glucose metabolism and homeostasis. FOXA2 plays a key role in maintaining glucose homeostasis by regulating the function of the glucostat system, which consists of the pancreas and other metabolic organs such as the liver and adipose tissue. These tissues monitor blood glucose levels and initiate appropriate responses to maintain balance. The figure was created using Biorender (https://biorender.com/).

FOXA2 is a key TF essential for maintaining systemic glucose homeostasis through its regulatory roles in the pancreas, liver, and adipose tissue (Fig. 5). It orchestrates gene expression programs that govern glucose sensing, insulin secretion, hepatic gluconeogenesis, and energy metabolism.

In pancreatic β-cells, FOXA2 regulates genes critical for glucose-stimulated insulin secretion, including PDX1, HNF family members, ABCC8, KCNJ11, and the glucose transporter GLUT2.^136^, ^137^, ^138^, ^139^ FOXA2 directly binds to the promoter regions of these genes, influencing the β-cell glucose threshold and insulin release. Loss- and gain-of-function studies confirm its role in modulating glucokinase and glucose transporter expression, shifting the glucose-stimulated insulin secretion response to lower glucose concentrations.^137^ The islet-specific glucose-6-phosphatase catalytic subunit-related protein (IGRP/G6PC2) exhibits selective expression in islet β-cells and significantly contributes to glucose homeostasis. Furthermore, it serves as a prominent autoantigen in type 1 diabetes.^140^ Chromatin immunoprecipitation assays have shown that the IGRP promoter binds several islet-enriched TFs, including Foxa2, indicating the metabolic regulatory role of Foxa2 in β-cells.^141^ In α-cells, FOXA2 influences glucagon biosynthesis. Its ablation results in reduced glucagon expression and lower plasma glucagon levels, although the secretion machinery remains functional.^82^ Thus, FOXA2 coordinates endocrine cell function to maintain glucose balance.

In addition to its pancreatic functions, FOXA2 plays a central role in fasting-induced hepatic gluconeogenesis. FOXA2 promotes gluconeogenesis by activating the expression of key genes in this metabolic pathway. A study on fasting mice demonstrated that FOXA2 recruits CREB (cyclic AMP response element-binding protein) and the glucocorticoid receptor (GR) to its target sites to initiate gluconeogenic gene expression.^142^ Furthermore, FOXA2 has been shown to recruit the ten-eleven translocation family member 3 (TET3) to the *Hnf4α* promoter 2 (P2), leading to promoter demethylation, transcriptional activation, and subsequent expression of *Hnf4α*. This, in turn, induces the expression of gluconeogenic enzymes such as phosphoenolpyruvate carboxykinase (*Pepck*) and glucose-6-phosphatase (*G6pc*), thereby enhancing hepatic glucose production.^143^ The importance of FOXA2 in this process is further supported in mouse models with hepatocyte-specific deletion of *Foxa2*, which exhibit impaired activation of *Pepck*, *G6pc*, and *tyrosine aminotransferase* (*Tat*) during fasting.^142^ In addition to gluconeogenesis, FOXA2 supports energy utilization over fat storage by enhancing the expression of genes involved in glucose uptake, glycolysis, and lipid metabolism. FOXA2 activity is modulated by insulin signaling. In response to feeding, insulin activates AKT2/PKB, which phosphorylates FOXA2 at threonine 156, resulting in its exclusion from the nucleus and inhibition of its transcriptional activity.^144^^,^^145^ This mechanism is thought to prevent FOXA2 from promoting gluconeogenesis and fatty acid oxidation in the fed state. Supporting this, constitutive nuclear localization of a non-phosphorylatable FOXA2 mutant in diabetic mouse models improves glucose tolerance, insulin sensitivity, hepatic fat metabolism, and energy expenditure. However, the proposed model of insulin-mediated nuclear exclusion remains controversial. Several studies report that FOXA2 remains nuclear in both fed and fasted states, including in wild-type and hyperinsulinemic ob/ob mice, and that its chromatin binding does not differ significantly between these metabolic states.^142^^,^^146^ This discrepancy underscores the need for further investigation into the context-specific regulation of FOXA2 and its precise contribution to diabetes pathogenesis.

FOXA2 also plays a critical role in regulating energy metabolism in adipose tissue. It promotes the expression of insulin-sensitizing and metabolic genes such as *Ucp2*, *Ucp3*, muscle pyruvate kinase, *hexokinase 2*, and *Glut4*, all of which are essential for maintaining energy balance and glucose homeostasis.^147^ FOXA2 also acts as a negative regulator of adipocyte differentiation by inducing the expression of preadipocyte factor-1 (*Pref-1*), thereby inhibiting adipocyte maturation and fat accumulation.^147^^,^^148^ Haploinsufficiency of *Foxa2* in mice leads to increased adiposity, particularly when exposed to a high-fat diet, accompanied by reduced energy expenditure and impaired metabolic flexibility.^147^ Furthermore, FOXA2 negatively regulates the *fat mass and obesity-associated (FTO)* gene, which is strongly linked to increased diabetes risk.^149^

## Deciphering FOXA2 contribution to diabetes pathogenesis

Given its wide-ranging metabolic functions, FOXA2 is increasingly implicated in diabetes. It regulates upstream TFs vital for β-cell development (*e.g.*, HNF4α, HNF1α, PDX1, HNF1β),^20^^,^^91^ mutations in which underlie monogenic forms of diabetes such as MODY1, MODY3, MODY4, and MODY5.^150^^,^^151^ Moreover, human developmental studies revealed that the FOXA2 deficiency leads to a significant reduction in the expression of several genes involved in other monogenic diabetes, including *NEUROD1*, *NGN3*, *PAX4*, *GATA6*, and *MNX1*.^62^ FOXA2 may also play a role in diabetes through its regulation of the expression of specific genes regulating the insulin secretory machinery of β-cells, including the ATP-dependent potassium channel subunits (*ABCC8* and *KCNJ11*) and the genes responsible for the secretory vesicles docking system,^84^^,^^137^ which is evidenced by the hyperinsulinemia hypoglycemia status observed in cases of Foxa2-specific ablation in β-cells.^61^ Pathogenic variants in the *ABCC8* and *KCNJ11* genes are frequently implicated in neonatal diabetes, especially in non-consanguineous families. These variants can also lead to later onset diabetes, identified as MODY12 in individuals with ABCC8 mutations and MODY 13 in those with KCNJ11 mutations.^152^^,^^153^ FOXA2 is a crucial transcriptional regulator of insulin sensitivity, affecting the expression of genes related to glucose and lipid metabolic pathways, which are often impaired in insulin resistance and type 2 diabetes (T2D).^144^ Given FOXA2's significant influence on the expression of crucial MODY-related genes, especially the same family member, it is reasonable to hypothesize that mutations in *FOXA2* may contribute to the development of MODY (Table 2). Early familial segregation studies and linkage association analysis have shown that genetic variation in the *FOXA2* gene is unlikely to be responsible for early-onset T2D and is not a common cause of MODY in French Caucasians and Japanese populations.^154^, ^155^, ^156^ However, several studies identified *FOXA2* mutations in different populations with MODY. Direct sequencing of the *FOXA2* gene in 68 Japanese subjects with MODY/early-onset diabetes identified one missense mutation, A328V, and seven polymorphisms.^157^ The proband, with the A328V mutation, is a non-obese 31-year-old man who experienced severe symptoms that led to a coma at the age of 5 years due to pronounced hyperglycemia with absolute insulin deficiency.^157^ Two other missense mutations, A86T and G114E, were identified in Japanese subjects with late-onset T2D, with A86T–HNF–3β variant showing approximately 85 % reduction in the transactivation activity.^158^ A genotyping study of a North Indian diabetic cohort revealed a strong association between the (TCC)n repeat polymorphism of the *FOXA2* gene and T2D. The identified A5 allele polymorphism at the (TCC)n locus is associated with higher fasting glucose levels and lower fasting insulin and C-peptide levels, suggesting that individuals homozygous for the A5 allele have a higher susceptibility to developing T2D.^159^ Recent genome-wide association studies (GWAS) found that the minor T-allele of FOXA2 rs1209523 is associated with lower fasting plasma glucose levels.^160^^,^^161^ In the same line, screening the genetic loci at genome-wide significance identified two other *FOXA2* SNPs, rs6048205 and rs6113722, associated with fasting glycemic trait and insulin resistance with the risk of T2D.^162^^,^^163^ A comprehensive genetic fine mapping study of 39 established T2D risk alleles in 27,206 cases of European ancestry has revealed a significant enrichment of variants within *FOXA2* binding sites at these susceptible loci.^164^ A patient with a novel de novo deletion of chromosome 20p11.2, which includes the *FOXA2* gene, has been documented to develop diabetes mellitus. By age 12, the patient exhibited hyperglycemia, an upper-normal glycated hemoglobin A1c (5.7 %), and impaired glucose tolerance.^139^ Specific deletion of *Foxa2* in mice β-cells has suggested it as a candidate gene for familial hyperinsulinism.^61^ In accordance, several human patients with *FOXA2* mutations showed the same congenital hyperinsulinism phenotype. The first human de novo missense *FOXA2* variant (p.S169P) was identified in a patient suffering from endodermal organ anomalies, syndromic hypopituitarism, and congenital hyperinsulinism.^165^ A similar phenotype with the hyperinsulinism was detected in individuals with de novo missense *FOXA2* variants (p.R257L, p.C222G, p.R256S, and p.N211K).^166^, ^167^, ^168^, ^169^ Mutations in the genes activated by *FOXA2,* such as *GCK*, *HNF4A*, *HNF1A*, *KCNJ11*, and *ABCC8,* are the most common cause of congenital hyperinsulinism.^170^ The early onset of hypoglycemia in patients could be explained by the hypo-glucagonemia observed in the mouse model.^64^ A patient with a heterozygous de novo nonsense c.616C > T (p.Q206X) mutation in *FOXA2* exon 2 was diagnosed with early neonatal severe hypoglycemia (0.27 mmol/L) with hyperinsulinism at age 3 months. The same patient was diagnosed later at age 5 years with central diabetes insipidus with pancreatic hypoplasia.^139^ Another patient with a heterozygous missense variant (c.633C > A, p.Asn211Lys) in the *FOXA2* gene recorded the same scenario with early hypoglycemic episode followed by hyperglycemia in early childhood at age 9.^169^ The early phenotype caused by a *FOXA2* defect transitions to insulin resistance and impaired glucose tolerance in later childhood. Therefore, it is crucial to have regular follow-up for patients with *FOXA2* variants to monitor for the development of diabetes. This biphasic phenotype is similar to the mutations observed in the MODY genes *HNF4A*, *HNF1A*, and *ABCC8*, where an initial phase of neonatal hyperinsulinemia hypoglycemia transitions into diabetes later in life.^171^, ^172^, ^173^ It has been suggested that the hyperinsulinemia in MODY3 is caused by the down-regulation of K-ATP channel genes, which precedes the failure of pancreatic β-cells in adolescence, causing diabetes.^174^ It is becoming more apparent that individuals with hyperinsulinism caused by dominant K-ATP channel mutations may see a decrease in symptoms with age, potentially leading to spontaneous remission, glucose intolerance, or diabetes.^175^

**Table 2:** *FOXA2* genetic variants associated with diabetes mellitus.

*FOXA2* genetic variation	Clinical symptoms	Reference
A328V	Severe symptoms of pronounced hyperglycemia with absolute insulin deficiency.	157
A86T	Reduction in the transactivation activity; late-onset type 2 diabetes mellitus	158
G114E	Reduction in the transactivation activity; late-onset type 2 diabetes mellitus	158
A5 allele polymorphism	Higher fasting glucose levels and lower fasting insulin and C-peptide levels	159
rs1209523 SNP	Lower fasting plasma glucose levels	160,161
rs6048205 SNP	Fasting glycemic trait and insulin resistance	162
rs6113722 SNP	Fasting glycemic trait and insulin resistance	163
De novo deletion of chromosome 20p11.2, including FOXA2 gene	The patient exhibited hyperglycemia, an upper-normal glycated hemoglobin A1c (5.7 %), and impaired glucose tolerance	139
p.S169P	Endodermal organ anomalies, syndromic hypopituitarism, and congenital hyperinsulinism.	165
p.R257L	An infant with severe neonatal hypoglycemia, hypopituitarism, and hyperinsulinism.	167
p.C222G	Congenital complex pituitary hormone deficiency (CPHD) with intestinal malrotation and anal atresia.	168
p.R256S	Hyperinsulinism	166, 167, 168, 169
p.N211K	Several malformations. Several hypoglycemic episodes at age 9 months	169
p.Q206X	At age 3 months: Early neonatal severe hypoglycemia with hyperinsulinism	139
	At age 5 years: Central diabetes insipidus with pancreatic hypoplasia	

## Conclusion and future perspectives

FOXA2 is a master transcriptional regulator at the intersection of developmental biology and metabolic homeostasis. Its role spans early endoderm specification to the establishment and maintenance of pancreatic endocrine identity, particularly within the glucagon-producing α-cells and insulin-secreting β-cells. FOXA2 coordinates complex gene regulatory networks that drive endocrine lineage commitment, modulate glucose-stimulated insulin secretion, and maintain glucose homeostasis through both pancreatic and hepatic pathways. Findings from conditional KO mouse models, hPSCs, and genetic studies in patients collectively underscore its indispensable function in pancreatic organogenesis and diabetes pathogenesis.

Despite significant advances, several gaps remain. The context-specific regulation of FOXA2, particularly its dynamic interaction with chromatin and other lineage-specific transcription factors, warrants further exploration. The precise mechanisms governing FOXA2's transcriptional activity in response to metabolic cues and tissue-specific roles remain incompletely understood, especially in human systems. Furthermore, while FOXA2 has emerged as a critical determinant of β-cell functionality, its role in the adaptation or failure of endocrine cells in the diabetic milieu requires deeper investigation.

Future studies should leverage single-cell transcriptomic and epigenomic profiling, genome-editing technologies, and organoid or islet-on-chip platforms to dissect FOXA2's regulatory logic in both physiological and disease contexts. From a therapeutic standpoint, targeting FOXA2-regulated pathways or modulating its activity could offer new avenues for regenerative strategies aimed at restoring islet function or preventing β-cell failure in diabetes. As a gatekeeper of islet identity and metabolic control, FOXA2 holds promise as both a biomarker and a potential target in precision medicine approaches for diabetes management.

## CRediT authorship contribution statement

**Ahmed K. Elsayed:** Writing – original draft, Methodology, Data curation, Conceptualization. **Yusra Manzoor:** Writing – original draft, Methodology, Data curation. **Essam M. Abdelalim:** Writing – review & editing, Supervision, Project administration, Funding acquisition, Conceptualization.

## Funding

This work was supported by a budget from 10.13039/100019475Sidra Medicine, Qatar (No. SDR400217).

## Conflict of interests

The authors declared no competing interests.
